# Exploring the Association Between *Torquetenovirus* Viral Load and Immunosuppressive Drug Exposure in Lung Transplantation

**DOI:** 10.3390/biom15040494

**Published:** 2025-03-27

**Authors:** Victor M. Mora, Emilio Rodrigo, David Iturbe-Fernández, Sheila Izquierdo, Sandra Tello, Adalberto Benito-Hernández, Maria Mar García-Saiz, David San Segundo, María Victoria Francia, Jose M. Cifrián

**Affiliations:** 1 Immunopathology Group, Respiratory Department, Marqués de Valdecilla University Hospital-IDIVAL, University of Cantabria, 39005 Santander, Spain; victormanuel.mora@scsalud.es (V.M.M.); david.iturbe@scsalud.es (D.I.-F.); sheila.izquierdo@scsalud.es (S.I.); sandra.tello@scsalud.es (S.T.); josemanuel.cifrian@scsalud.es (J.M.C.); 2 Immunopathology Group, Nephrology Department, Marqués de Valdecilla University Hospital-IDIVAL, University of Cantabria, 39005 Santander, Spain; adalberto.benito@scsalud.es; 3 Clinical Pharmacology Department, Marqués de Valdecilla University Hospital-IDIVAL, 39005 Santander, Spain; mmar.garcia@scsalud.es; 4 Immunopathology Group, Immunology Department, Marqués de Valdecilla University Hospital-IDIVAL, University of Cantabria, 39005 Santander, Spain; david.sansegundo@scsalud.es; 5 Infectious Diseases and Clinical Microbiology Group, Marqués de Valdecilla University Hospital-IDIVAL, University of Cantabria, 39005 Santander, Spain; mvictoriafrancia@scsalud.es

**Keywords:** acute rejection, immune response, immunosuppressive drugs, infection, monitoring, mycophenolic acid, tacrolimus, *Torquetenovirus*, lung transplantation

## Abstract

To improve lung transplant recipient (LungTx) outcome, it would be of great interest to measure the net state of immunosuppression to avoid both infection and rejection. Measurement of *Torquetenovirus* load (TTV load) has been proposed as a biomarker to monitor solid organ transplantation, but its relationship with immunosuppressive drugs, particularly mycophenolic acid (MPA), is not well understood. We performed a prospective study of 53 LungTx, measuring TTV load before transplantation, at week 3, and at month 3. Tacrolimus and MPA doses and levels were recorded, and an area under the MPA curve (AUC-MPA) was calculated at the third month. LungTx in the fourth quartile of TTV load at the third week and the third month exhibited a low risk of acute rejection (OR 0.113, 95% CI 0.013–0.953, *p* = 0.045) and a high risk of opportunistic infection from month 3 to 6 (OR 15.200, 95% CI 1.525–151.511, *p* = 0.020), respectively. TTV load was weakly related to tacrolimus trough level at month 3 (rho = 0.283, *p* = 0.040). Neither MPA blood levels nor AUC-MPA were related to TTV load, although only patients with a reduction in MPA dose from month 1 to 3 showed a smaller increase in TTV load (0.86, IQR 2.58 log10 copies/mL vs. 2.26, IQR 3.02 log10 copies/mL, *p* = 0.026). In conclusion, TTV load in LungTx is only partially related to exposure to immunosuppressive drugs. Other variables, such as inflammation, immunosenescence, and frailty, may influence the overall level of immunosuppression and TTV load.

## 1. Introduction

Lung transplantation (LungTx) is the optimal treatment for patients with end-stage respiratory diseases. The number of LungTx procedures has increased worldwide, with improved outcomes [1]. Nevertheless, patients undergoing LungTx have a high mortality and morbidity rate, primarily due to the elevated risk of infection and rejection [2]. In the solid organ transplantation field, post-transplant infection, particularly with regard to opportunistic infections, has been associated with overimmunosuppression, while the emergence of acute rejection has been linked to underimmunosuppression. Routine monitoring of lung allograft status is achieved through respiratory function tests and transbronchial biopsies, while immunological monitoring involves the measurement of trough levels of calcineurin inhibitors (CNI). However, it has been established that the latter better reflects the CNI toxicity than the risk of rejection [3,4]. Consequently, there is an imperative for novel instruments that facilitate enhanced assessment of the comprehensive immune status in transplant patients.

A promising biomarker of immune status is the presence of *Torquetenovirus* (TTV) in the blood. In LungTx recipients, an elevated TTV blood load (TTV load) measured at different times has been associated with a higher risk of infection and a lower risk of rejection [5,6,7,8]. Similarly, a recent meta-analysis has reported an elevated risk of infection (OR: 1.16, 95% CI: 1.03–1.32; HR: 1.05, 95% CI: 0.97–1.14) and a diminished risk of rejection (OR: 0.90, 95% CI: 0.87–0.94; HR: 0.74, 95% CI: 0.71–0.76) per 1 log TTV load increase in all types of solid organ transplantations [9]. Some studies have indicated that TTV load levels are elevated in patients undergoing more intensive immunosuppression therapy [6,7,10,11,12,13,14] and with higher calcineurin inhibitor trough levels [5,6,7], but other studies have not confirmed these findings [8,10,15,16,17,18]. The relationship with the trough levels of immunosuppressants remains to be elucidated, and the cumulative effect of exposure to immunosuppressive therapy has yet to be studied. Two studies in kidney transplant recipients have found an association between discontinuation of mycophenolic acid (MPA) and a decrease in TTV load [19,20], and Strassl et al. reported that TTV load is higher in kidney transplant recipients receiving MPA doses above 1.5 g [14]. However, no specific study has analyzed the relationship between blood MPA levels and TTV load in any solid organ transplantation. Our main objective was to analyze, for the first time in lung transplant recipients, the relationship between TTV viral load and continuous and timely exposure to immunosuppressive drugs, including not only CNI but also MPA. We hypothesized that a higher TTV load is associated with a higher exposure to tacrolimus and MPA in LungTx.

## 2. Materials and Methods

### 2.1. Study Design and Patients’ Information

A prospective study was conducted, incorporating all patients admitted to the LungTx waiting list at our center from September 2019 to November 2022 who were willing to participate. The study was conducted in accordance with the Declaration of Helsinki and approved by the Regional Ethics Committee at the institution (reference number: PI20/01710, approval date 22 December 2020). Our center is accredited by the National Transplant Organization of the government of Spain as a reference center for carrying out lung transplants within the national public health system. All organs were obtained from deceased donors. No patient on the waiting list expired prior to LungTx, while two patients died in the initial month post-transplant and were consequently excluded from the study. All patients received a double lung transplant between February 2021 and April 2023. HLA eplet mismatch load was calculated to establish donor and recipient compatibility. The HLA Matchmaker 3.1 software (available at http://www.epitopes.net/downloads.html, accessed on 1 April 2024) was utilized to assess eplet matching.

Recipient, donor, and transplant-relevant data and outcomes were prospectively recorded from the electronic medical records (Table 1). Primary graft dysfunction (PGD) was defined and graded according to ISHLT criteria [21]. A transbronchial biopsy is routinely performed in our center in the third week post-transplant. Acute cellular rejection post-transplantation was defined and graded based on the ISHLT Working Formulation [22]. All patients received induction therapy with basiliximab a minimum of two hours prior to un-clamping the first pulmonary artery and on the fourth day. The maintenance immunosuppressive therapy comprised mycophenolate mofetil (1000 mg/12 h), corticosteroids, and tacrolimus, with target trough blood levels between 12 and 15 ng/mL during the initial six months post-transplantation. The standard anti-infective prophylaxis regimen administered was piperacillin-tazobactam for the initial 4 days and trimethoprim-sulfamethoxazole 400/80 mg/24 h for the prevention of Pneumocystis jirovecii infection. Nebulized amphotericin B was administered for six months as prophylaxis against Aspergillus, and valganciclovir for six months for intermediate-risk CMV matches (pre-transplant IgG-positive recipient) and 12 months for high-risk matches (IgG-positive donor and IgG-negative recipient). In high-risk matches, specific anti-CMV immunoglobulins were also administered.

### 2.2. Immunosuppressive Drugs Monitoring

Whole blood concentrations (ng/mL) of tacrolimus were determined by chemiluminescent microparticle immunoassay using the Architect iSystem (CMIA; Abbott Laboratories, Abbott Park, North Chicago, IL, USA). All tacrolimus levels were recorded up to day 90. The variability of tacrolimus blood levels was estimated by the coefficient of variation (CV), calculated according to the following equation:

CV = (σ/µ) × 100,

where σ is the standard deviation and µ is the mean tacrolimus concentration of all available samples [23]. The percentage of time in the therapeutic range (TTR) (12 to 15 ng/mL) up to months 1 and 3 was calculated using the Rosendaal method [24]. The calculation of cumulative exposure to tacrolimus at month 3 was performed by determining the area under the curve of all tacrolimus trough concentrations, extending from the transplant date up to month 3 [25].

Trough blood concentrations of mycophenolic acid (MPA) in human plasma (mg/L) were quantified by homogeneous enzyme-linked immunosorbent assay (Emit 2000 Mycophenolic Acid Assay; Siemens, Munich, Germany) at months 1 and 3. The full mycophenolic acid area under the curve (AUC-MPA) was calculated at month 3 using an abbreviated procedure with blood samples taken at time 0, 30 min, and 2 h after drug intake, according to the procedure previously reported by Pawinski et al. The regression equation for AUC 0–12 h estimation that gave the best performance for this model was [26]:

7.75 + 6.49.C_0h_ + 0.76.C_0.5h_ + 2.43.C_2h_

AUC-MPA was not performed in three patients who refused the procedure and one patient with previous MPA withdrawal.

### 2.3. TTV Measurement

Samples for measurement of TTV load were taken at the following times: while the patient was on the waiting list (baseline); at week 3, coinciding with the surveillance transbronchial biopsy; and at day 90, where previous reports have identified the post-transplant peak of TTV load that is stable up to month 6 [8,17]. Free viral DNA was purified from 400 mL of plasma from all specimens using the QIAamp MinElute Virus Spin Kit Cat. #57704 (Qiagen GmbH, Hilden, Germany) as specified by the manufacturer. The presence and viral load of TTV in the samples were determined in duplicate using a previously described TaqMan (TM)-PCR assay for human TTV APP2XDMP (ThermoFisher, Life Technologies, Paisley, UK) standardized and checked by Maffi et al. and Pistello et al. in a StepOnePlus Real-Time PCR System (AB Applied Biosystems, Singapore) [27,28,29]. This assay is based on the specific amplification of a highly conserved viral segment in the untranslated region of TTV, which has the potential for sensitive and specific detection of all TTV genotypes present in GenBank [27]. The procedures employed for the quantification of copy number, in addition to the assessment of specificity, sensitivity, intra- and inter-assay precision, and reproducibility, have been previously delineated [28,29]. We used as a positive control a 143 bp PCR fragment from the same untranslated region of the *TTV genome* (NC_015783.1) that was amplified using the primers *TTVSen* (5′GTGCCGTAGGTGAGTTTA3′) and *TTVAntisenseL* (5ATGGACCGGCGGTCTCCACGG3′) and cloned into the pCR™2.1 cloning vector (TA Cloning™ Kit, # K202040, ThermoFisher, Invitrogen, Carlsbad, CA, USA). The resulting plasmid was then purified with a QIAprep Spin Miniprep Kit, #2710 (Qiagen GmbH, Hilden, Germany), and quantified using a Nanodrop 2000C spectrophotometer, Thermo Fisher Scientific # ND-2000C (Thermo Fisher Scientific, Wilmington, DE, USA). The standard curve was established with the points A = 1.0 × 10^12^ copies, B = 1.0 × 10^10^ copies, C = 1.0 × 10^8^ copies, D = 1.0 × 10^6^ copies, E = 1.0 × 10^4^ copies, F = 1.0 × 10^2^ copies, G = 1.0 copies, and H = 0 copies [18]. The lower limit of sensitivity was established at 1.0 × 10³ viral genomes per ml of plasma sample. A recent comparison and validation of this protocol was conducted using the commercial TTV R-GENE^®^ kit (bioMérieux, Craponne, France) [30]. ΔLogTTV was calculated as the difference between the logarithm of TTV load at month 3 and at month 1.

### 2.4. Statistical Analysis

Continuous variables were expressed as mean standard deviation if normally distributed or as median and interquartile range (IQR) if non-normally distributed. Categorical variables were described as relative frequencies. The relationship between dichotomous variables was analyzed using the chi-square test. The relationship between TTV load and ΔLogTTV, as well as continuous variables, was explored using Spearman’s rank correlation coefficient. The Wilcoxon rank test was used to compare TTV loads at different time points, and the Mann–Whitney U test was used to compare TTV load differences among dichotomous variables. The ability of TTV load to discriminate infection and rejection was analyzed by constructing receiver operating characteristic (ROC) curves. Univariate logistic regression analysis was used to analyze the relationship between TTV load and infection and rejection. A *p*-value less than 0.05 was considered statistically significant. Statistical analyses were performed with SPSS, version 22.0 (SPSS, Inc., Chicago, IL, USA).

## 3. Results

**Figure 1:**
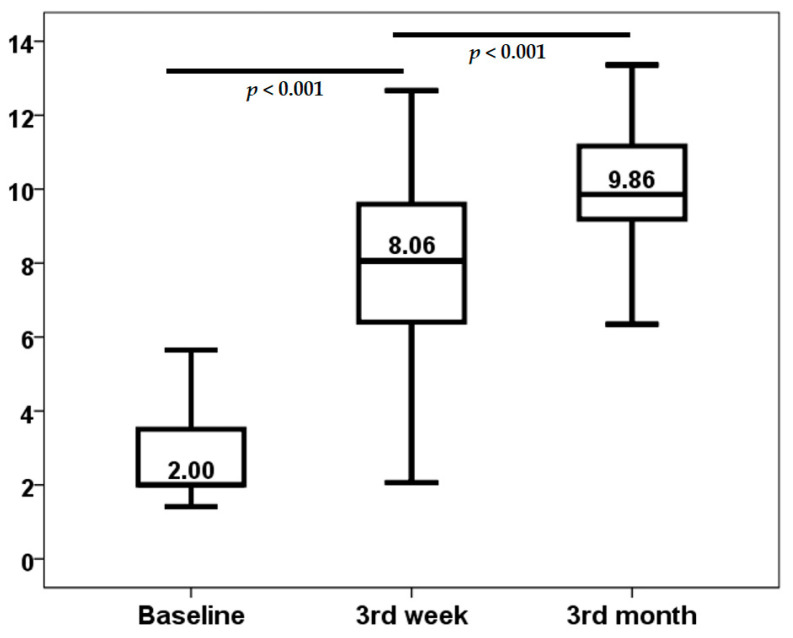
Kinetics of TTV load (log10 copies/mL).

The demographic and clinical characteristics of the patient cohort are delineated in Table 1. The study encompassed 53 lung transplant recipients, who were monitored during the initial year post-transplantation. A notable increase in median TTV load was observed, escalating from a baseline level of 2.00 log10 copies/mL (interquartile range [IQR]: 1.59 log10 copies/mL) to a median value of 8.06 log10 copies/mL (IQR: 3.23 log10 copies/mL) by the third week post-transplantation. This increase was statistically significant (*p* < 0.001). A similar trend was observed when the data was examined from the third week to the third month post-transplant (*p* < 0.001) (Figure 1). The baseline TTV load demonstrated no correlation with recipient age (rho = 0.061, *p* = 0.666), body mass index (BMI) (rho = −0.145, *p* = 0.300), serum creatinine (rho = 0.181, *p* = 0.194), and Lung Allocation Score (LAS) (rho = 0.068, *p* = 0.628). Furthermore, baseline TTV load did not differ according to recipient gender (*p* = 0.985), IPF as underlying disease (*p* = 0.162), previous smoking habit (*p* = 0.359), previous hypertension (*p* = 0.543), or diabetes diagnosis (*p* = 0.564).

PGD developed in 10 patients (18.9%), with severe dysfunction occurring in 5 (9.4%). Neither baseline (*p* = 0.584) nor third-week TTV loads (*p* = 0.544) were higher in patients with severe PGD. In addition, no correlation was found between baseline (rho = −0.215, *p* = 0.121) or third week (rho = 0.170, *p* = 0.223) TTV loads and PGD severity.

**Figure 2:**
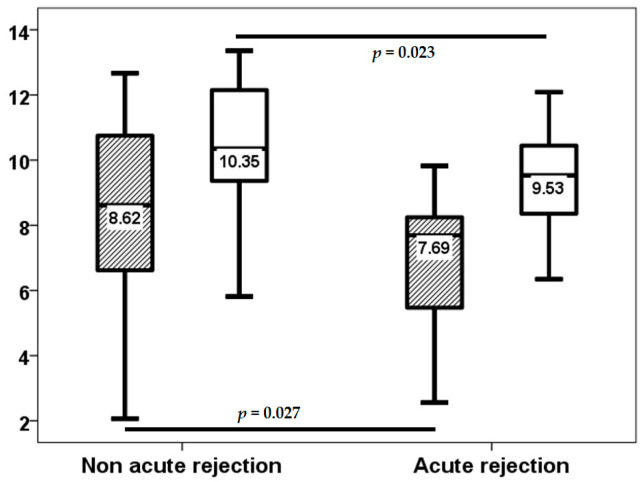
TTV load (log10 copies/mL) at the third week (stripped bars) and third month (white bars) in patients with and without third-week acute rejection.

**Figure 3:**
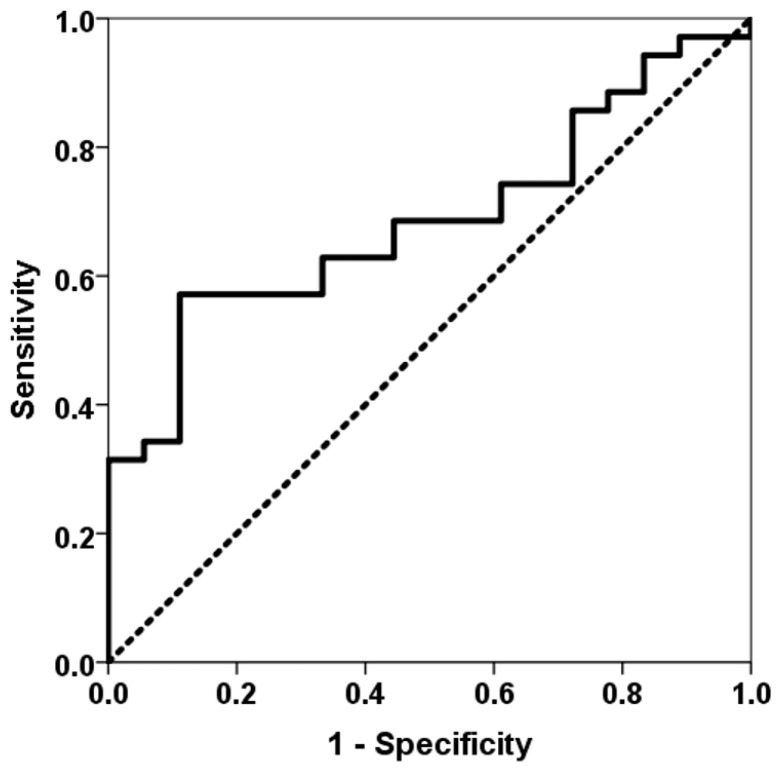
ROC curve of TTV load for predicting acute rejection at the third week. The solid line represents the ROC curve (AUC = 0.687). The dotted line represents the reference ROC curve with an AUC of 0.50.

As indicated by the third-week surveillance biopsy, acute rejection was observed in 18 (34%) patients. The severity of acute cellular rejection in the vascular compartment was categorized as follows: grade A1 in 3 patients, grade A2 in 12 patients, grade A3 in 2 patients, and grade A4 in 1 patient. Notably, only 3 patients experienced rejection from month 1 to 3, and 1 patient from month 9 to 12. The third week TTV load (8.62, IQR 4.45 log10 copies/mL vs. 7.69, IQR 2.92 log10 copies/mL, *p* = 0.027) and the third month TTV load (10.35, IQR 3.00 log10 copies/mL vs. 9.53, IQR 2.60 log10 copies/mL, *p* = 0.023) were lower in LungTx with acute rejection at the third week (Figure 2). Conversely, baseline TTV load did not differ between patients with early rejection (*p* = 0.157). TTV load at the third week was found to have the capacity to differentiate between patients with acute rejection, with an area under the curve (AUC) of 0.687 (95% CI 0.546–0.829, *p* = 0.027) (Figure 3), whereas the AUC-ROC curve for baseline TTV load was not significant (0.610, 95% CI 0.450–0.771, *p* = 0.192). Subsequent regression analysis revealed that TTV load in the third week was associated with a reduced risk of acute rejection (OR 0.785, 95% CI 0.622–0.991, *p* = 0.042). No other variable was found to be associated with the risk of acute rejection. Patients in the fourth quartile of the TTV load in the third week exhibited a reduced rate of acute rejection (43% vs. 8%, *p* = 0.021) and a very low risk of acute rejection (OR 0.113, 95% CI 0.013–0.953, *p* = 0.045) according to logistic regression analysis. Due to the limited number of acute rejection episodes beyond the first month, no further analysis was conducted to ascertain the relationship between TTV load at month 3 and acute rejection.

**Figure 4:**
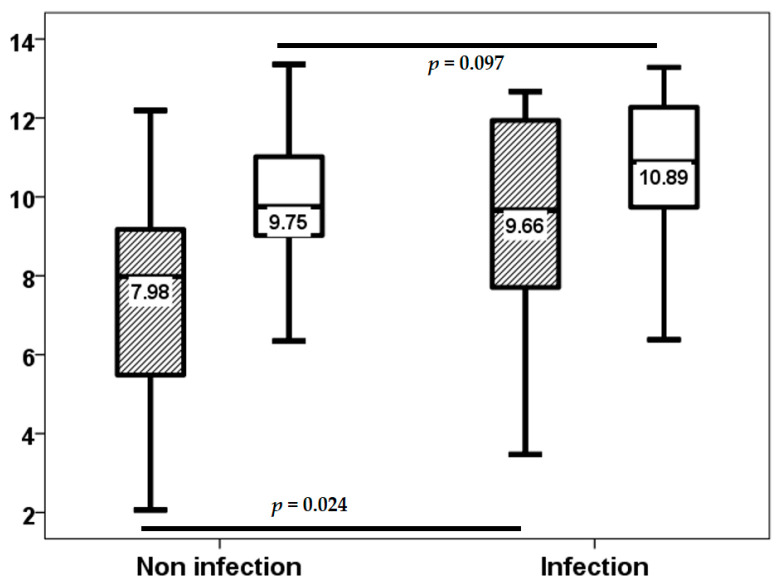
TTV load (log10 copies/mL) at the third week (stripped bars) and third month (white bars) in patients with and without infection from months 3 to 6.

**Figure 5:**
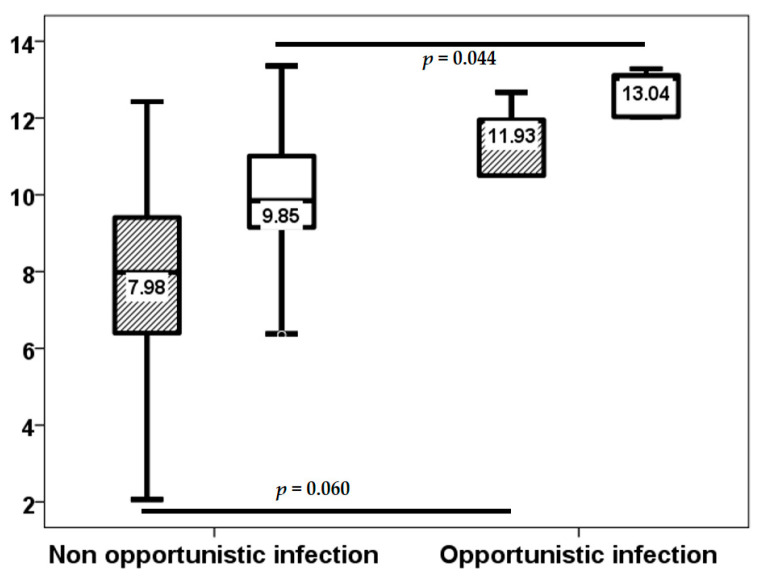
TTV load (log10 copies/mL) at the third week (stripped bars) and third month (white bars) in patients with and without opportunistic infection from month 3 to 6.

In the initial three-week period, 18 (34%) of the 54 lung transplant recipients experienced at least one infection episode, of which only 2 (3.8%) were classified as opportunistic infections. Subsequent to this, through to the third month, 12 (22.6%) patients developed an infection episode, with only one opportunistic infection. From months 3 to 6, the number of patients with at least one infection episode increased to 15 (28.3%), with only 5 (9.4%) of these being opportunistic infections. Baseline TTV load exhibited no statistically significant differences between patients with and without infection up to the third week (2.00, IQR 2.86 log10 copies/mL vs. 2.73, IQR 1.36 log10 copies/mL, *p* = 0.984).TTV load at the third week did not relate to a higher risk of infection (8.24, IQR 3.28 log10 copies/mL vs. 7.70, IQR 3.24 log10 copies/mL, *p* = 0.318) from this point to the month 3, third week TTV load (7.98, IQR 3.75 log10 copies/mL vs. 9.66, IQR 4.35 log10 copies/mL, *p* = 0.024), but not third month TTV load (9.75, IQR 2.13 log10 copies/mL vs. 10.89, IQR 2.60 log10 copies/mL, *p* = 0.097) related to a higher infection rate from month 3 to 6 (Figure 4). third month TTV loads (9.85, IQR 1.88 vs. 13.04, IQR 3.22 log10 copies/mL, *p* = 0.044) related to a higher opportunistic infection rate from month 3 to 6, and the third week (7.98, IQR 3.02 log10 copies/mL vs. 11.93, IQR 5.32 log10 copies/mL, *p* = 0.060) was not significantly related (Figure 5).

Due to the significant overlap in TTV loads between patients with and without infection, as demonstrated in Figure 4 and Figure 5, we proceeded to analyze the relationship between the fourth quartiles of TTV load at week 3 and at month 3. Patients in the fourth quartile of TTV load at the third week exhibited a higher infection rate (17.5% vs. 61.5%, *p* = 0.002) and opportunistic infection from month 3 to 6 (2.5% vs. 30.8%, *p* = 0.002). Although patients in the fourth quartile of TTV load at third month did not demonstrate a significantly higher rate of infection (23.1% vs. 42.9%, *p* = 0.159), the rate of opportunistic infection was significantly higher (2.6% vs. 28.6%, *p* = 0.004). The fourth quartile of TTV load at week 3 was associated with an elevated risk of infection (OR 7.543, 95% CI 1.891–30.083, *p* = 0.004) and opportunistic infection (OR 17.333, 95% CI 1.724–174.285, *p* = 0.015) from month 3 to 6. The fourth quartile of TTV load at the third month did not relate to a higher risk of infection (OR 2.500, 95% CI 0.685–9.121, *p* = 0.165). However, a higher risk of opportunistic infection was observed from month 3 to 6 (OR 15.200, 95% CI 1.525–151.511, *p* = 0.020).

**Figure 6:**
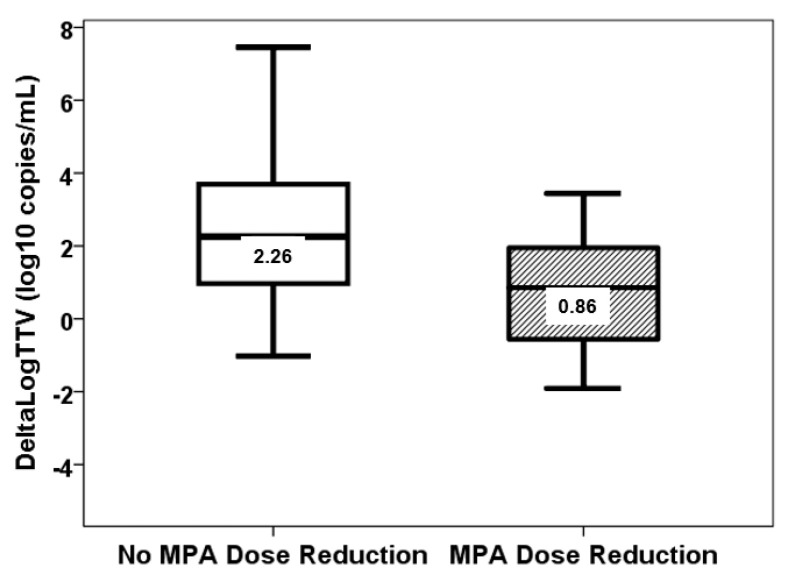
ΔLogTTVM13 (log10 copies/mL) in patients with (stripped bar) and without (white bar) reduction of MPA doses from month 1 to 3 (*p* = 0.026).

Neither the third week nor the third month TTV load correlated with age, BMI, serum creatinine, LAS, and HLA mismatches (Table 2 and Table 3). In the third week, TTV load was not different according to gender (*p* = 0.231), IPF (*p* = 0.344), hypertension (*p* = 0.083), diabetes (*p* = 0.918), previous smoking habit (*p* = 0.901), CMV serologic mismatch (*p* = 0.265), or PGD (*p* = 0.117). The third month TTV load was not different according to gender (*p* = 0.761), IPF (*p* = 0.524), hypertension (*p* = 0.555), diabetes (*p* = 1.000), previous smoking habit (*p* = 0.321), CMV serologic mismatch (*p* = 0.146), or PGD (*p* = 0.256). TTV load at the third week was not associated with any pharmacological variable (Table 2). TTV load at third month is only related to the trough level of tacrolimus at month 3 (Table 3). In this study, no patient received MPA doses above 1440 mg/day. The association between ΔLogTTVM13 and the reduction in MPA doses between months 1 and 3 was found to be statistically significant (rho = −0.336, *p* = 0.014). However, no such association was observed between ΔLogTTVM13 and the changes in tacrolimus doses (rho = 0.011, *p* = 0.938), tacrolimus trough levels (rho = −0.208, *p* = 0.135), MPA trough levels (rho = −0.058, *p* = 0.685), or prednisone doses (rho = −0.112, *p* = 0.425) between months 1 and 3. A reduction in MPA dose was observed in 39 patients from month 1 to 3, and these patients exhibited a diminished ΔLogTTV (0.86, IQR 2.58 log10 copies/mL vs. 2.26, IQR 3.02 log10 copies/mL, *p* = 0.026) (Figure 6).

## 4. Discussion

In accordance with the findings of preceding researchers, a correlation was identified between TTV load and acute rejection in the cohort of lung transplant recipients under investigation [5,7,8]. This correlation has been documented in not only lung transplant recipients but also in recipients of other solid organ transplants, including those of the liver, kidney, and heart [9]. The area under the curve (AUC) of the ROC curve for TTV load in discriminating acute rejection was 0.687, which is similar to the reported values of 0.73 and 0.67 for Doberer et al. and Strassl et al., respectively [13,31]. As the level of risk of rejection increased by one log10 unit, the probability of rejection decreased by 21%, a finding that is consistent with previous reports that ranged from 10 to 50% [9,13,30,31,32]. Of interest, patients with a TTV load in the upper quartile were found to have a significantly reduced risk of acute rejection (89%).

We also found that the low TTV loads that increased the risk of rejection at the third week were maintained more than 2 months later despite the intense steroid therapy. Even though patients with rejection had received high-dose steroid treatment in the third week, as is the recommended standard practice, TTV load values remained significantly lower at the third month in patients who had suffered acute rejection. Previous studies have reported that low-TTV loads preceded the development of acute rejection around 60–90 days in LungTx and from 14 to 43 days in kidney transplantation, but none have reported the TTV load evolution after rejection therapy [8,13,31]. Reineke et al. reported that 31 kidney transplant recipients treated with high-dose corticosteroid pulses as anti-rejection therapy exhibited a significant increase in TTV load from biopsy to 30 and 90 days. However, it is important to note that these results cannot be extrapolated to lung transplant recipients due to the lower TTV loads exhibited by kidney transplant recipients throughout all the following [33]. Our finding suggests that steroid therapy solves the rejection (1-month follow-up biopsies showed improvement of the infiltrates up to A0 or A1) through intragraft mechanisms but without increasing the net state of immunosuppression as measured by TTV load. This finding would need to be confirmed and analyzed in depth in further studies.

The present study did not identify a clear relationship between TTV and the risk of infection. Neither baseline nor third week TTV loads were found to be associated with the rate of infection throughout the first month or from the third week to the third month. By contrast, lung transplant recipients with high TTV loads at the third month were found to suffer significantly more opportunistic infections beyond the third month. The risk increased close to tenfold for every one-unit rise in the third-month TTV load, and this impact was similar to that previously reported by Strassl et al. [14]. These discrepancies regarding the usefulness of TTV viral load among the variables that have been most commonly analyzed (acute rejection and infection) have also been written about by other authors. As indicated by van Rijn et al., TTV load has been demonstrated to be a useful tool in the establishment of the immunosuppressive state, which is associated with an elevated risk of rejection, though not of infection, in kidney transplant recipients [32]. One potential explanation for this observation is that acute rejection is a more clearly defined endpoint, whereas the definition of infection is more variable and is partially influenced by non-immune factors, such as the reactivation of latent pathogens or exposure to new environmental or donor pathogens [9].

Whilst earlier research has indicated a correlation between gender and age and TTV load [10,14], this relationship has not been substantiated by our research or that of other authors [7,34]. In contrast to the findings of Doorenbos et al., no such correlation was observed between viral load and previous smoking habits, although it should be noted that no patient was admitted to the LungTx waiting list as an active smoker [35]. Furthermore, the previously reported association between CMV serostatus and TTV load was not identified in the present study [10,14,36].

In order to understand the exact relationship between immunosuppressive treatment and TTV viral load, we studied in depth the overall exposure to different immunosuppressive drugs. With regard to induction treatment, previous studies had demonstrated that induction treatment initially led to a decrease in TTV load during the initial weeks, followed by an increase in viral load. This initial decrease was attributed to the specific effect of induction on T lymphocytes, where TTV replicates, with a subsequent increase in relation to a more intense immunosuppression that persists for the first few months after transplantation in patients with lymphocyte-depleting induction [12,36,37]. All our lung transplant recipients received basiliximab; consequently, it was not possible to analyze the impact of induction on TTV load. Furthermore, no relationship was identified between prednisone dose and TTV viral load.

In relation to the administration of tacrolimus doses, earlier research has yielded contradictory results. In congruence with the present study, Jaksch et al. and Görzer et al. identified a correlation between individual tacrolimus trough blood levels and TTV load [6,7]. Conversely, other researchers did not observe this association [8,10,12,14,15,16,17,37]. The reported correlation between TTV loads and tacrolimus levels has always been weak, which may explain the discrepancies observed among different studies [6,7]. In view of the weak or non-existent correlation between isolated levels of tacrolimus and TTV load, the hypothesis was tested of whether continuous exposure would prove to be more strongly related. To this end, the cumulative exposure to tacrolimus was analyzed by means of different methods. First, the mean CNI trough levels from transplantation up to months 1 and 3 were measured. Secondly, the cumulative exposure to tacrolimus was calculated according to the method proposed by Rodriguez-Peralvarez, which had previously demonstrated that liver transplant recipients exposed to higher cumulative tacrolimus doses throughout the first three months showed a higher risk of cancer and therefore a greater overimmunosuppressive status [25]. Thirdly, we analyzed the variability in exposure to tacrolimus (time in therapeutic range or coefficient of variability) that had been related to a higher risk of toxicity and poor outcome in patients with lung transplants and which can be used as a surrogate marker of lack of adherence to CNI therapy [24,38]. Neither cumulative exposure to tacrolimus nor variability influenced the TTV viral load values in our study. These findings suggest that continuous exposure to tacrolimus does not influence TTV levels, whereas isolated point levels may have an influence. This conclusion is supported by the study recently reported by Regele et al. The authors reported that the TTV load decreased significantly 60 days after decreasing the tacrolimus doses and trough levels. Unfortunately, the same study revealed that elevating tacrolimus doses and levels resulted in a non-significant trend of increasing TTV load two months later [39]. The results available to date preclude the establishment of a precise conclusion on the manner and timing of the optimization of the overall immunosuppression status of a patient, as measured by the TTV viral load, through the modification of CNI levels. The results of three ongoing randomized controlled trials are expected to provide a more accurate knowledge of the relationship between tacrolimus exposure and TTV load [40,41,42]. The VIGILung is a two-center, ongoing, controlled trial that includes 144 LungTx subjects. In this trial, the tacrolimus target range will be adjusted based on TTV load in the active group, as opposed to the conventional tacrolimus blood level monitoring employed in the control group [40].

In addition, a prospective exploration was conducted into the relationship between TTV load and MPA exposure. This investigation entailed the analysis of MPA levels in blood samples collected at the third week and third month. Furthermore, the area under the curve (AUC) of MPA was also analyzed, as this is the gold standard for determining MPA exposure. To this end, a 3-point curve with a demonstrated good performance was selected to estimate MPA-AUC [26]. Because the most important immunosuppressive activity of tacrolimus is exerted at the lymphocyte level, lower MPA levels would be expected to be associated with lower viral loads and a diminished risk of infection. Recently, Benning et al. and Regele et al. have reported a 50% reduction in TTV load following MPA withdrawal [19,20]. The present study has failed to establish a correlation between MPA-AUC, MPA trough levels or doses, and TTV load. Conversely, any decrease in MPA dose between months 1 and 3 was reflected in a reduced increase in viral load between months 1 and 3. Consequently, variations in TTV load are contingent on alterations in the MPA dose, rather than on its blood levels.

Although we hypothesized that a higher TTV burden, and thus a higher level of overall immunosuppression, was associated with higher tacrolimus and MPA exposure in LungTx, our study showed that this relationship was weak, suggesting that there should be other factors influencing immunocompetence/immunosuppression status. For example, cytomegalovirus infection is known to promote rejection by disrupting transplant tolerance [43] but also increases the risk of subsequent infections as CMV downregulates the innate and adaptive arms of the immune system [44]. Therefore, viral infections might not only increase the degree of immunosuppression but also the relationship between immunosuppression and related events, such as rejection and infections. In the non-transplant population, other non-pharmacological factors have been identified as influencing the immunosuppression status determined by TTV viral load. In a cross-sectional study involving 900 healthy controls and 86 patients with ischemic heart disease, a high TTV load was associated with higher plasma levels of proinflammatory cytokines and shorter telomeres compared to those with low TTV viremia [45]. In the MARK-AGE study, high TTV viremia was also associated with physical frailty [46]. Therefore, inflammation, immunosenescence, and frailty may be some of these nonpharmacological factors that influence the immunosuppression status and should be taken into account to properly assess variations in TTV viral load after solid organ transplantation.

The most significant limitation of our study was its single-center design and the fact that it included a relatively small number of patients, which limits the generalizability of its conclusions. Conducting multicenter studies with larger numbers of patients and undertaking in-depth analyses of the relationship between TTV load and exposure to the various immunosuppressive drugs would be necessary to obtain stronger conclusions. However, it was a prospective study in which cumulative exposure to tacrolimus and MPA blood levels were studied for the first time. Another potential limitation is that the study utilized a non-standardized in-house PCR technique. Nevertheless, Kulifaj et al. demonstrated the agreement between in-house and standardized techniques and that both methods were useful for measuring TTV [47]. Finally, budgetary constraints meant that TTV viral load was only measured at three time points (pre-transplant, third week, and third month). Evidently, a greater number of determinations would have enabled more frequent monitoring and a more in-depth analysis of the relationship between TTV viral load and the different post-transplant events. However, it should be noted that other authors have also followed similar monitoring schedules [37] that cover the early post-transplant period, in which a higher number of immunosuppression-related events occur.

## 5. Conclusions

This study has demonstrated that lung transplant recipients with low early TTV loads are at an elevated risk of allograft rejection in the initial weeks following transplantation. However, the correlation between high TTV loads and an augmented infection risk was only substantiated at the third month. While TTV load can be used as a surrogate marker of the net immunosuppressive status, no clear relationship was found between TTV load and the cumulative exposure to tacrolimus. Furthermore, the correlation between tacrolimus drug levels and TTV load was weak. Of interest, changes in MPA doses, but not MPA blood levels, were associated with changes in TTV viral load. Consequently, we can speculate that a comprehensive evaluation of the immunosuppression status post-transplantation, as determined by TTV viral load, should encompass additional non-pharmacological factors, such as inflammation, immunosenescence, and frailty. The information obtained from the three ongoing randomized trials will be of great interest in facilitating a comprehensive understanding of two key elements: firstly, the usefulness of the TTV load measurement as a marker of immunosuppression level; and secondly, its modification by immunosuppressive drugs.

## Figures and Tables

**Table 1:** Main patient characteristics.

Main Patient Characteristics	
Number of patients	53
Recipient age (years)	59.5 ± 8.3
Recipient gender (male)	45.3%
BMI (kg/m^2^)	24.4 ± 2.9
Underlying disease	
IPF	39.6%
COPD	50.9%
Previous smoking habit	75.5%
Pretransplant Diabetes	9.4%
Pretransplant Hypertension	32.1%
Serum creatinine (mg/dL)	0.71 ± 0.15
Donor age ≥ 60 years	34.0%
Lung Allocation Score	34.0 ± 3.4
HLA-ABCDRDQ mismatch	54 ± 17
Donor/Recipient CMV Serologic Mismatch	18.9%
Second lung ischemia time (min)	376 ± 78
Pulmonary Graft Dysfunction 2/3	9.4%
Third week: acute rejection	18 (34.0%)
Third week Prednisone dose (mg)	22.4 ± 4.2
TTR 12–15 ng/mL at the third week (%)	28 ± 19%
Mean tacrolimus level at third week (ng/mL)	19.1 ± 34.8
Coefficient of variability at third week	52.0 ± 9.3
Mean MPA level at third week (mg/L)	2.9 ± 1.6
Third month Prednisone dose (mg)	19.7 ± 1.2
TTR 12–15 ng/mL at third month (%)	38 ± 17%
Mean tacrolimus level at third month (ng/mL)	12.4 ± 2.4
Coefficient of variability at third month	42.8 ± 8.0
Tacrolimus cumulative exposure at third month (ng∙day/mL)	1092 ± 105
Mean MPA level at third month (mg/L)	3.0 ± 1.7
AUC-MPA at third month (µg·h/mL)	58.2 ± 23.2

BMI = body mass index, IPF = idiopathic pulmonary fibrosis, COPD = chronic obstructive pulmonary disease, HLA-ABCDRDQ mismatch = number of mismatches in HLA class I A, B and C and in class II DR and DQ, CMV = cytomegalovirus, TTR = time in therapeutic range, MPA = mycophenolic acid, and AUC = area under the curve.

**Table 2:** Spearman correlation analysis between continuous variables and TTV load at the third week.

	rho	*p*
Recipient age (years)	0.098	0.485
BMI (kg/m^2^)	−0.090	0.524
Serum creatinine (mg/dL)	0.005	0.971
Lung Allocation Score	0.002	0.991
HLA-ABCDRDQ eplet mismatches	−0.216	0.120
third week Prednisone dose (mg)	−0.194	0.163
TTR 12–15 ng/mL at third week (%)	0.067	0.632
Mean tacrolimus levels up to month 1	0.090	0.520
Mean tacrolimus level at third week (ng/mL)	0.043	0.758
Coefficient of variability at the third week	0.043	0.761
Mean MPA level at third week (mg/L)	0.023	0.872

BMI = body mass index, HLA-ABCDRDQ mismatch = number of mismatches in HLA class I A, B and C and in class II DR and DQ, TTR = time in therapeutic range, MPA = mycophenolic acid.

**Table 3:** Spearman correlation analysis between continuous variables and TTV load at the third month.

	rho	*p*
Recipient age (years)	−0.014	0.919
BMI (kg/m^2^)	−0.007	0.958
Serum creatinine (mg/dL)	0.111	0.429
Lung Allocation Score	0.020	0.887
HLA-ABCDRDQ eplet mismatches	0.051	0.718
third month Prednisone dose (mg)	0.000	1.000
TTR 12–15 ng/mL at third month (%)	−0.151	0.281
Mean tacrolimus levels up to month 3	0.169	0.226
Mean tacrolimus level at third month (ng/mL)	0.283	0.040
Coefficient of variability at third month	0.059	0.676
Tacrolimus cumulative exposure at third month (ng∙day/mL)	0.201	0.152
Mean MPA level at third month (mg/L)	0.060	0.668
AUC-MPA at third month (µg·h/mL)	−0.082	0.578

BMI = body mass index, HLA-ABCDRDQ mismatch = number of mismatches in HLA class I A, B and C and in class II DR and DQ, TTR = time in therapeutic range, MPA = mycophenolic acid, and AUC = area under the curve.

## Data Availability

The data presented in this study are available on request from the corresponding author.

## Abbreviations

The following abbreviations are used in this manuscript:

AUC	Area under the curve
BMI	Body mass index
CI	Confidence interval
CMV	Cytomegalovirus
CNI	Calcineurin inhibitors
COPD	Chronic obstructive pulmonary disease
CV	coefficient of variation
HLA	Human leukocyte antigens
IPF	Idiopathic pulmonary fibrosis
IQR	Interquartile range
ISHLT	International Society for Heart and Lung Transplantation
LAS	Lung Allocation Score
LungTx	Lung transplant recipients
MPA	Mycophenolic acid
OR	Odds ratio
PGD	Primary graft dysfunction
ROC	Receiver operating characteristic
TTR	Time in the therapeutic range
TTV Load	*Torquetenovirus* load
ΔLogTTV	Difference between the logarithm of TTV load at month 3 and at month 1

## References

[1] Christie J.D., Van Raemdonck D., Fisher A.J. (2024). Lung Transplantation. N. Engl. J. Med..

[2] McCort M., MacKenzie E., Pursell K., Pitrak D. (2021). Bacterial infections in lung transplantation. J. Thorac. Dis..

[3] Napoli C., Benincasa G., Fiorelli A., Strozziero M.G., Costa D., Russo F., Grimaldi V., Hoetzenecker K. (2024). Lung transplantation: Current insights and outcomes. Transpl. Immunol..

[4] Andrews L.M., Li Y., De Winter B.C.M., Shi Y.Y., Baan C.C., Van Gelder T., Hesselink D.A. (2017). Pharmacokinetic considerations related to therapeutic drug monitoring of tacrolimus in kidney transplant patients. Expert Opin. Drug Metab. Toxicol..

[5] De Vlaminck I., Khush K.K., Strehl C., Kohli B., Luikart H., Neff N.F., Okamoto J., Snyder T.M., Cornfield D.N., Nicolls M.R. (2013). Temporal response of the human virome to immunosuppression and antiviral therapy. Cell.

[6] Görzer I., Haloschan M., Jaksch P., Klepetko W., Puchhammer-Stöckl E. (2014). Plasma DNA levels of *Torque teno virus* and immunosuppression after lung transplantation. J. Heart Lung Transplant..

[7] Jaksch P., Kundi M., Görzer I., Muraközy G., Lambers C., Benazzo A., Hoetzenecker K., Klepetko W., Puchhammer-Stöckl E. (2018). *Torque Teno Virus* as a Novel Biomarker Targeting the Efficacy of Immunosuppression After Lung Transplantation. J. Infect. Dis..

[8] Frye B.C., Bierbaum S., Falcone V., Köhler T.C., Gasplmayr M., Hettich I., Dürk T., Idzko M., Zissel G., Hengel H. (2019). Kinetics of *Torque Teno Virus*-DNA Plasma Load Predict Rejection in Lung Transplant Recipients. Transplantation.

[9] van Rijn A.L., Roos R., Dekker F.W., Rotmans J.I., Feltkamp M. (2023). *Torque teno virus* load as marker of rejection and infection in solid organ transplantation—A systematic review and meta-analysis. Rev. Med. Virol..

[10] Schiemann M., Puchhammer-Stöckl E., Eskandary F., Kohlbeck P., Rasoul-Rockenschaub S., Heilos A., Kozakowski N., Görzer I., Kikić Ž., Herkner H. (2017). *Torque Teno Virus* Load-Inverse Association With Antibody-Mediated Rejection After Kidney Transplantation. Transplantation.

[11] Burra P., Masier A., Boldrin C., Calistri A., Andreoli E., Senzolo M., Zorzi M., Sgarabotto D., Guido M., Cillo U. (2008). *Torque Teno Virus*: Any pathological role in liver transplanted patients?. Transpl. Int..

[12] Focosi D., Macera L., Pistello M., Maggi F. (2014). *Torque Teno virus* viremia correlates with intensity of maintenance immunosuppression in adult orthotopic liver transplant. J. Infect. Dis..

[13] Strassl R., Doberer K., Rasoul-Rockenschaub S., Herkner H., Görzer I., Kläger J.P., Schmidt R., Haslacher H., Schiemann M., Eskandary F.A. (2019). *Torque Teno Virus* for Risk Stratification of Acute Biopsy-Proven Alloreactivity in Kidney Transplant Recipients. J. Infect. Dis..

[14] Strassl R., Schiemann M., Doberer K., Görzer I., Puchhammer-Stöckl E., Eskandary F., Kikic Ž., Gualdoni G.A., Vossen M.G., Rasoul-Rockenschaub S. (2018). Quantification of *Torque Teno Virus* Viremia as a Prospective Biomarker for Infectious Disease in Kidney Allograft Recipients. J. Infect. Dis..

[15] Solis M., Velay A., Gantner P., Bausson J., Filipputtu A., Freitag R., Moulin B., Caillard S., Fafi-Kremer S. (2019). *Torquetenovirus* viremia for early prediction of graft rejection after kidney transplantation. J. Infect..

[16] Ruiz P., Martínez-Picola M., Santana M., Muñoz J., Pérez-Del-Pulgar S., Koutsoudakis G., Sastre L., Colmenero J., Crespo G., Navasa M. (2019). *Torque Teno Virus* Is Associated With the State of Immune Suppression Early After Liver Transplantation. Liver Transpl..

[17] Görzer I., Jaksch P., Kundi M., Seitz T., Klepetko W., Puchhammer-Stöckl E. (2015). Pre-transplant plasma *Torque Teno virus* load and increase dynamics after lung transplantation. PLoS ONE.

[18] Cañamero L., Benito-Hernández A., González E., Escagedo C., Rodríguez-Vidriales M., García-Saiz M.D.M., Valero R., Belmar L., de Cos M.A., Francia M.V. (2023). *Torque Teno Virus* Load Predicts Opportunistic Infections after Kidney Transplantation but Is Not Associated with Maintenance Immunosuppression Exposure. Biomedicines.

[19] Regele F., Heinzel A., Hu K., Raab L., Eskandary F., Faé I., Zelzer S., Böhmig G.A., Bond G., Fischer G. (2022). Stopping of Mycophenolic Acid in Kidney Transplant Recipients for 2 Weeks Peri-Vaccination Does Not Increase Response to SARS-CoV-2 Vaccination-A Non-randomized, Controlled Pilot Study. Front. Med..

[20] Benning L., Morath C., Kühn T., Bartenschlager M., Kim H., Beimler J., Buylaert M., Nusshag C., Kälble F., Reineke M. (2022). Humoral response to SARS-CoV-2 mRNA vaccination in previous non-responder kidney transplant recipients after short-term withdrawal of mycophenolic acid. Front. Med..

[21] Snell G.I., Yusen R.D., Weill D., Strueber M., Garrity E., Reed A., Christie J.D. (2017). Report of the ISHLT Working Group on Primary Lung Graft Dysfunction. part I: Definition and grading—A 2016 Consensus Group statement of the International Society for Heart and Lung Transplantation. J. Hear Lung Transplant..

[22] Stewart S., Fishbein M.C., Snell G.I., Berry G.J., Boehler A., Burke M.M., Glanville A., Gould F.K., Magro C., Marboe C.C. (2007). Revision of the 1996 working formulation for the standardization of nomenclature in the diagnosis of lung rejection. J. Heart Lung Transplant..

[23] Rodrigo E., San Segundo D., Fernández-Fresnedo G., López-Hoyos M., Benito A., Ruiz J.C., de Cos M.A., Arias M. (2016). Within-Patient Variability in Tacrolimus Blood Levels Predicts Kidney Graft Loss and Donor-Specific Antibody Development. Transplantation.

[24] Ensor C.R., Iasella C.J., Harrigan K.M., Morrell M.R., Moore C.A., Shigemura N., Zeevi A., McDyer J.F., Venkataramanan R. (2018). Increasing tacrolimus time-in-therapeutic range is associated with superior one-year outcomes in lung transplant recipients. Am. J. Transpl..

[25] Rodríguez-Perálvarez M., Colmenero J., González A., Gastaca M., Curell A., Caballero-Marcos A., Sánchez-Martínez A., Di Maira T., Herrero J.I., Almohalla C. (2022). Chronic immunosuppression, cancer Spanish consortium. Cumulative exposure to tacrolimus and incidence of cancer after liver transplantation. Am. J. Transplant..

[26] Pawinski T., Luszczynska P., Durlik M., Majchrzak J., Baczkowska T., Chrzanowska M., Sobiak J., Glyda M., Kuriata-Kordek M., Kaminska D. (2013). Development and validation of limited sampling strategies for the estimation of mycophenolic acid area under the curve in adult kidney and liver transplant recipients receiving concomitant enteric-coated mycophenolate sodium and tacrolimus. Ther. Drug Monit..

[27] Maggi F., Pifferi M., Fornai C., Andreoli E., Tempestini E., Vatteroni M., Presciuttini S., Marchi S., Pietrobelli A., Boner A. (2003). TT virus in the nasal secretions of children with acute respiratory diseases: Relations to viremia and disease severity. J. Virol..

[28] Maggi F., Fornai C., Vatteroni M.L., Siciliano G., Menichetti F., Tascini C., Specter S., Pistello M., Bendinelli M. (2001). Low prevalence of TT virus in the cerebrospinal fluid of viremic patients with central nervous system disorders. J. Med. Virol..

[29] Pistello M., Morrica A., Maggi F., Vatteroni M.L., Freer G., Fornai C., Casula F., Marchi S., Ciccorossi P., Rovero P. (2001). TT virus levels in the plasma of infected individuals with different hepatic and extrahepatic pathology. J. Med. Virol..

[30] Görzer I., Haupenthal F., Maggi F., Gelas F., Kulifaj D., Brossault L., Puchhammer-Stöckl E., Bond G. (2023). Validation of plasma Torque Teno viral load applying a CE-certified PCR for risk stratification of rejection and infection post kidney transplantation. J. Clin. Virol..

[31] Doberer K., Schiemann M., Strassl R., Haupenthal F., Dermuth F., Görzer I., Eskandary F., Reindl-Schwaighofer R., Kikić Ž., Puchhammer-Stöckl E. (2020). *Torque teno virus* for risk stratification of graft rejection and infection in kidney transplant recipients-A prospective observational trial. Am. J. Transplant..

[32] van Rijn A.L., Wunderink H.F., Sidorov I.A., de Brouwer C.S., Kroes A.C., Putter H., de Vries A.P., Rotmans J.I., Feltkamp M.C. (2021). *Torque teno virus* loads after kidney transplantation predict allograft rejection but not viral infection. J. Clin. Virol..

[33] Reineke M., Morath C., Speer C., Rudek M., Bundschuh C., Klein J.A.F., Mahler C.F., Kälble F., Nusshag C., Beimler J. (2024). Dynamics of *torque teno virus* load in kidney transplant recipients with indication biopsy and therapeutic modifications of immunosuppression. Front. Med..

[34] Takemoto A.Y., Okubo P., Saito P.K., Yamakawa R.H., Watanabe M.A., Veríssimo da Silva Junior W., Borelli S.D., Bedendo J. (2015). *Torque teno virus* among dialysis and renal- transplant patients. Braz. J. Microbiol..

[35] Doorenbos C.S.E., Jonker J., Hao J., Gore E.J., Kremer D., Knobbe T.J., de Joode A.A.E., Sanders J.S.F., Thaunat O., Niesters H.G.M. (2023). Smoking, Alcohol Intake and *Torque Teno Virus* in Stable Kidney Transplant Recipients. Viruses.

[36] Fernández-Ruiz M., Albert E., Giménez E., Ruiz-Merlo T., Parra P., López-Medrano F., San Juan R., Polanco N., Andrés A., Navarro D. (2019). Monitoring of alphatorquevirus DNA levels for the prediction of immunosuppression-related complications after kidney transplantation. Am. J. Transplant..

[37] Reineke M., Speer C., Bundschuh C., Klein J.A.F., Loi L., Sommerer C., Zeier M., Schnitzler P., Morath C., Benning L. (2024). Impact of induction agents and maintenance immunosuppression on *torque teno virus* loads and year-one complications after kidney transplantation. Front. Immunol..

[38] Gallagher H.M., Sarwar G., Tse T., Sladden T.M., Hii E., Yerkovich S.T., Hopkins P.M., Chambers D.C. (2015). Erratic tacrolimus exposure, assessed using the standard deviation of trough blood levels, predicts chronic lung allograft dysfunction and survival. J. Heart Lung Transplant..

[39] Regele F., Haupenthal F., Doberer K., Görzer I., Kapps S., Strassl R., Bond G. (2024). The kinetics of *Torque Teno virus* plasma load following calcineurin inhibitor dose change in kidney transplant recipients. J. Med. Virol..

[40] Gottlieb J., Reuss A., Mayer K., Weide K., Schade-Brittinger C., Hoyer S., Jaksch P. (2021). Viral load-guided immuno suppression after lung transplantation (VIGILung)-study protocol for a randomized controlled trial. Trials.

[41] Haupenthal F., Rahn J., Maggi F., Gelas F., Bourgeois P., Hugo C. (2023). A multicentre, patient- and assessor-blinded, non-inferiority, randomised and controlled phase II trial to compare standard and *torque teno virus*-guided immuno suppression in kidney transplant recipients in the first year after transplantation: TTVguideIT. Trials.

[42] Kapps S., Haupenthal F., Bond G. (2024). *Torque Teno Virus*-guided monitoring of immunosuppressive therapy. Nephrol. Dial. Transplant..

[43] Yu S., Dangi A., Burnette M., Abecassis M.M., Thorp E.B., Luo X. (2021). Acute murine cytomegalovirus disrupts established transplantation tolerance and causes recipient allo-sensitization. Am. J. Transplant..

[44] Freeman R.B. (2009). The ‘indirect’ effects of cytomegalovirus infection. Am. J. Transplant..

[45] Giacconi R., Piacenza F., Maggi F., Bürkle A., Moreno-Villanueva M., Mancinelli L., Spezia P.G., Novazzi F., Drago Ferrante F., Minosse C. (2024). Association Between TTV Viremia, Chronic Inflammation, and Ischemic Heart Disease Risk: Insights From MARK-AGE and Report-Age Projects. J. Gerontol. A Biol. Sci. Med. Sci..

[46] Giacconi R., Laffon B., Costa S., Teixeira-Gomes A., Maggi F., Macera L., Spezia P.G., Piacenza F., Bürkle A., Moreno-Villanueva M. (2023). Association of Torquetenovirus Viremia with Physical Frailty and Cognitive Impairment in Three Independent European Cohorts. Gerontology.

[47] Kulifaj D., Durgueil-Lariviere B., Meynier F., Munteanu E., Pichon N., Dubé M., Joannes M., Essig M., Hantz S., Barranger C. (2018). Development of a standardized real time PCR for *Torque teno viruses* (TTV) viral load detection and quantification: A new tool for immune monitoring. J. Clin. Virol..

